# K_ATP_ Channel Opener Diazoxide Prevents Neurodegeneration: A New Mechanism of Action via Antioxidative Pathway Activation

**DOI:** 10.1371/journal.pone.0075189

**Published:** 2013-09-11

**Authors:** Noemí Virgili, Pilar Mancera, Blanca Wappenhans, Georgina Sorrosal, Knut Biber, Marco Pugliese, Juan F. Espinosa-Parrilla

**Affiliations:** 1 Neurotec Pharma S.L., Bioincubadora PCB-Santander, Parc Científic de Barcelona, Barcelona, Spain; 2 Advancell, S.A, Barcelona, Spain; 3 Department of Psychiatry and Psychotherapy, University of Freiburg, Freiburg, Germany; 4 Departament de Ciències Fisiològiques I, Institut d’Investigacions Biomèdiques August Pi i Sunyer (IDIBAPS), Universitat de Barcelona and Centro de Investigación Biomédica en Red sobre Enfermedades Neurodegenerativas (CIBERNED), Barcelona, Spain; Oregon Health & Science University, United States of America

## Abstract

Pharmacological modulation of ATP-sensitive potassium channels has become a promising new therapeutic approach for the treatment of neurodegenerative diseases due to their role in mitochondrial and cellular protection. For instance, diazoxide, a well-known ATP-sensitive potassium channel activator with high affinity for mitochondrial component of the channel has been proved to be effective in animal models for different diseases such as Alzheimer’s disease, stroke or multiple sclerosis. Here, we analyzed the ability of diazoxide for protecting neurons front different neurotoxic insults *in vitro* and *ex vivo*. Results showed that diazoxide effectively protects NSC-34 motoneurons from glutamatergic, oxidative and inflammatory damage. Moreover, diazoxide decreased neuronal death in organotypic hippocampal slice cultures after exicitotoxicity and preserved myelin sheath in organotypic cerebellar cultures exposed to pro-inflammatory demyelinating damage. In addition, we demonstrated that one of the mechanisms of actions implied in the neuroprotective role of diazoxide is mediated by the activation of Nrf2 expression and nuclear translocation. Nrf2 expression was increased in NSC-34 neurons *in vitro* as well as in the spinal cord of experimental autoimmune encephalomyelitis animals orally administered with diazoxide. Thus, diazoxide is a neuroprotective agent against oxidative stress-induced damage and cellular dysfunction that can be beneficial for diseases such as multiple sclerosis.

## Introduction

ATP-sensitive potassium (K_ATP_) channels are ubiquitous ion channels coupling cell metabolism to electrical activity by regulating the potassium flow across the cell membrane. These channels act as energy sensors of ATP production and are believed to regulate various physiological functions, such as muscle contraction and insulin secretion, by coupling ATP/ADP balance to membrane potential [1], [2]. In the central nervous system (CNS), K_ATP_ channels are described to be important in glucose sensing in different areas including the midbrain (substantia nigra), pons (locus coeruleus) and medulla (nucleus of the solitary tract) [3], [4]. K_ATP_ channels also play a role in neurosecretion at nerve terminals. [5], [6].

K_ATP_ channels are also present at the mitochondrial inner membrane (mito-K_ATP_). Mito-K_ATP_ channels participate in the regulation of mitochondrial volume, ionic homeostasis, pH gradient and membrane potential [7]. Furthermore, their activity is related to electronic transport, metabolic energy, reactive oxygen species (ROS) production and mitochondrial welfare and can be an interesting target for neuroprotective therapies [8], [9]. Mito-K_ATP_ channels are identified in liver mitochondria [10], heart [11], kidney [12], skeletal muscle [13] and brain [14], where it has been estimated to contain six to seven times more mito-K_ATP_ concentration than liver or heart. Activation of these channels allows potassium ions to flow into mitochondria which results in depolarization and may initiate neuronal preconditioning [15].

Diazoxide (7-chloro-3-methyl-4H-1,2,4-benzothiadiazine 1,1-dioxide), a well-known small molecule that opens K_ATP_ channels with high affinity for mito-K_ATP_ channels, has been reported to cause cardio and neuroprotection. This effect has been mainly attributed to its ability to decrease calcium overload from the extracellular space [16], but more important, due to its activity in regulating mitochondrial homeostasis [17]. Diazoxide demonstrated neuroprotective effects in cerebral ischemia-reperfusion injury diminishing levels of reactive oxygen species, decreasing DNA oxidative damage, and inhibiting caspase-dependent and -independent apoptotic pathways while preserving mitochondrial structure [18]–[20]. The opening of K_ATP_ channels by diazoxide can modulate the nigro-striatal pathway reducing akinesia in a dose-dependent manner in a reserpine-treated rat model of Parkinson disease (PD) [21]. In addition, neuroprotective effects on dopaminergic neurons by regulating glial reactivity though mito-K_ATP_ activation has been also described [22], [23]. Other authors demonstrated that diazoxide is able to protect neurons against β-amyloid toxicity, reduce protein aggregates and tau hyperphosphorilation and to improve cognitive function and learning behavior [24]–[26]. Thus, K_ATP_ channels and its pharmacological modulation are recognized as potential targets and neuroprotective treatments of several neurodegenerative diseases such as ischemia, PD or Alzheimer’s diseases (AD) [26]–[29].

We previously reported that oral administration with diazoxide ameliorated clinical signs on myelin oligodendrocyte glycoprotein peptide (MOG35-55)-induced experimental autoimmune encephalomyelitis (EAE), a murine model of multiple sclerosis (MS). At histological level diazoxide elicited a significant reduction in myelin and axonal loss accompanied by a decrease in glial activation and neuronal damage without affecting the number of infiltrating lymphocytes positive for CD3 and CD20 in the spinal cord [30]. Thus, these results demonstrated a novel action of diazoxide for preventin brain damage in neuroinflammation. MS pharmacotherapy is dominated by immunomodulatory drugs, which were developed to reduce the incidence and intensity of new inflammatory lesions by limiting the activity of immune cells without acting directly on preventing CNS damage. However, in the last years, the therapeutic landscape for MS is rapidly changing and the development of drugs with primarily CNS neuroprotective effects is currently a pharmaceutical reality. Among other neuroprotective strategies, the activation of cellular self-defense against oxidative stress, particularly the transcription factor nuclear factor (erythroid-derived 2)-related factor 2 (Nrf2)-mediated pathway has been postulated as a novel therapeutic approach for the treatment of neurodegenerative disease [31], [32]. For instance, the activation of Nrf2 nuclear translocation has been described as the main mechanism of action involved in the beneficial effect of fumaric esters for the treatment of MS [33]. Nrf2 mediates the activation of detoxifying phase II enzymes via mitochondrial oxidative stress signaling [34], [35] where mito-K_ATP_ channels function play a crucial role [36]. This pathway may thus play a critical role for cellular protection in an environment of oxidative stress and may represent an interesting therapeutic target in neurodegenerative diseases.

Consequently, the aims of the present study were: (a) to study the protective effects of diazoxide against excitotoxicity, oxidative, pro-inflammatory and demyelinating insults, and (b) to analyze the effects of diazoxide in activating the anti-oxidative self-defense in neurons, proposing a novel neuroprotective mechanism of action based on Nrf2 activation.

## Materials and Methods

### Ethics Statement

Animal were handled according to European legislation (86/609/EU) and procedures were approved by the Ethics and Scientific Committees of the University of Barcelona and registered at the “Departament d'Agricultura, Ramaderia i Pesca, Generalitat de Catalunya, Spain”.

### Reagents

Diazoxide was purchased from Sigma-Aldrich (St. Louis, MO, USA). Stock solutions (50 mM) of diazoxide were prepared in dimethyl sulfoxide (DMSO, Sigma-Aldrich). Solutions for cell treatment were prepared by diluting stock solutions in culture media immediately before being added to the cells (DMSO concentration: 0.5%). Solutions for animal treatment were prepared by diluting stock solution in water every day of the treatment (DMSO concentration: 0.3%).

### Mice

Female C57BL/6J mice, 8 to 10 weeks of age, were purchased from Charles River (Sulzfeld, Germany) and maintained on a 12∶12 h light:dark cycle, with standard chow and water freely available.

### EAE induction and treatment

EAE was induced by immunization with > 95% pure synthetic MOG35-55 peptide (rat MOG35-55, MEVGWYRSPFSRVVHLYRNGK; EspiKem Srl, Florence, Italy). Mice were injected subcutaneously at one side of the flank with 100 µL solution containing 150 µg of rat MOG in complete Freund's adjuvant (Sigma-Aldrich) and 5 mg/mL Mycobacterium tuberculosis H37Ra (Difco Laboratories, Detroit, MI, USA). Mice also received intraperitoneal injections of 150 ng pertussis toxin (Sigma-Aldrich) in 100 µL phosphate-buffered saline (PBS) immediately after MOG injection and 48 h later.

To study Nrf2 activation in diazoxide treated EAE mice, two different administration protocols were performed: in the first one, treatment began on the first day of EAE induction (preventive) whereas the second one started in the chronics phase, when the EAE clinical score was ≥ 1 (appearance of clinical signs, therapeutic). The MOG-immunized mice were administered either 0.8 mg/kg diazoxide (treated group) or diluent (0.3% DMSO in water, vehicle group) for 30 or 15 days by oral gavage, respectively.

### NSC-34 cell culture experiments

Motoneuronal NSC-34 cell line (Cellutions Biosystems Inc, Cedarlane, Ontario, Canada) was a gift from Dr. Villoslada (IDIBAPS, Barcelona, Spain). Cells were cultured at 37°C and 5% CO2 in Dulbecco’s modified Eagle’s medium (DMEM) supplemented with 10% fetal bovine serum (FBS), 1% penicillin/streptomycin and 0.04 mM L-glutamine (all from Invitrogen, Eugene, OR, USA). To differentiate NSC-34 cells to a motoneuronal and glutamate-responsive phenotype, DMEM was replaced by DMEM/Ham’s F12 (Invitrogen) supplemented with 1% FBS, 1% penicillin/streptomycin and 1% modified Eagle’s medium nonessential amino acids (Sigma-Aldrich) [37]. NSC-34 cells were seeded at low density (3×10^4^ cells/ml) in 24-well plates and were used 72 h after seeding for the toxicity assays. For the treatments, control wells contained the same final concentration of vehicle as the compound-containing wells (0.5% DMSO).

#### Glutamate toxicity assay

NSC-34 cells were allowed to differentiate for 8 weeks under reduced serum conditions and then seeded in 24-well plates at a density of 3×10^4^ cells/ml for the following experiment. Glutamate (Sigma-Aldrich) was dissolved in culture medium and added to cultures at concentration of 10 mM for 24 h. Cell treatment with 100 µM diazoxide started 2 h before glutamate exposure. Cell viability was measured by the 3-[4,5-dimethylthiazol-2-yl]-2,5-diphenyltetrazolium bromide (MTT) assay.

#### Hydrogen peroxide exposures

To induce oxidative stress, hydrogen peroxide (H_2_O_2_) was added to final concentration of 0.2 mM (Stock 30%, Sigma-Aldrich). NSC-34 cells were exposed to H_2_O_2_ for 30 min at 37°C. Then the medium was removed and replaced with fresh medium for 24 h. Cells were treated with 100 µM diazoxide 2 h before H_2_O_2_ injury and during 24 h after. Cell viability was measured by the MTT assay.

#### BV2 conditioned medium

To test inflammatory damage, conditioned medium (CM) from unstimulated and Lipopolysacharide (LPS, Sigma-Aldrich) plus gamma Interferon (IFN-γ, Sigma-Aldrich) activated BV2 microglia was used to damage neuron cells. BV2 cells were seeded and activated as previously described [30]. Cell medium was collected 24 h after activation and then used to replace NSC-34 medium. NSC-34 viability was measured 24 h after BV2 CM exposure by MTT assay. Diazoxide (100 µM) was tested in two ways: in the first one, it was added to BV2 cells prior to activation and in the second one, drug was added to NSC-34 cells before CM addition.

#### 2,2'-azobis-2-methyl-propanimidamide, dihydrochloride (AAPH) exposure

AAPH, a free radical generating azo compound, was used to induce oxidative stress. NSC-34 cells were pretreated for 2 h with different concentrations of diazoxide (100, 10, 1 and 0.1 µM). Then, the medium was removed and replaced with fresh medium containing AAPH 9 mM (Sigma-Aldrich). Cells were then maintained with the treatment for 24 h at 37°C and cell viability was measured by MTT assay.

### 3-(4,5-Dimethyl-2-thiazolyl)-2,5-diphenyl-2H-tetrazolium bromide reduction method (MTT)

MTT reduction assay was used as an indicator of cell viability. MTT (Sigma-Aldrich) was added to a well at a final concentration of 0.5 mg/mL. After MTT incubation at 37°C, DMSO was added and cells were gently resuspended. Absorbances at 560 and 620 nm were recorded with a microplate reader (BioTek ELX800, BioTek Instruments Inc., Vermont, USA). Mean values of at least three independent wells from three experiments were calculated, and cell survival was expressed as percentage MTT-positive cells compared with untreated control cells.

### Immunofluorescence for Nrf2 in spinal cord

Histological procedures for coronal spinal cord EAE mice sections followed the same methodology previously described [30]. The following antibodies were used: anti-NeuN (1:100, Millipore, Bedford, MA, USA) and anti-Nrf2 (1:75, Santa Cruz Biotechnology). The secondary antibodies used were Alexa®488 and 596 (from 1∶2000 to 1∶1000, Molecular Probes, Invitrogen, Eugene, OR, USA). Double immunofluorescent staining was performed after citrate Heat-Induced Epitope Retrieval (HIER) of the samples as previously described [30].

Images were captured using both wide field microsope Leica AF7000 and SP1 confocal microscope (Leica Microsystems GmbH, Wetzlar, Germany), located at the Parc Científic de Barcelona, Barcelona, Spain.

### Isolation of nuclear and total proteins for Nrf2 Western Blot

Nrf2 level was determined in nuclear protein extracts from NSC-34 cell cultures 24 h after treatments, using 3 wells from 6-well plates for each experimental condition. Nuclear protein extraction from cell cultures were performed as previously described [38]. Protein concentration was determined by the Lowry assay (Total Protein kit micro-Lowry, Sigma-Aldrich). For total protein extraction for *in vivo* western blot spinal cord samples were first sonicated at 4°C in RIPA buffer (Sigma-Aldrich), and protease inhibitor cocktail Complete (Roche Diagnostics, Basel, Switzerland). After 30 min of incubation on ice, samples were centrifuged at 5000 rpm for 5 min at 4°C and the supernatants were collected. Protein concentration was determined by the Lowry assay as above.

### Western blot

Western blot analyses for total and nuclear extracts (40 µg of denatured protein) were performed as described [30]. Briefly, polyvinylidene difluoride membrane (Millipore) with protein extracts were incubated with primary antibodies: polyclonal rabbit anti-Nrf2 (1:300, Santa Cruz Biotechnology), polyclonal goat anti-lamin B (1:7500, Santa Cruz Biotechnology) and monoclonal mouse anti-actin (1:100000, Sigma-Aldrich) and secondary antibodies donkey anti-rabbit (1:5000, Amersham, Buckinghamshire, UK), donkey anti-goat (1:2000, Santa Cruz Biotechnology), and donkey anti-mouse (1:5000, Santa Cruz Biotechnology) respectively. Western blotting detection was performed with ECL Plus (Amersham). The membranes were then exposed to the camera and the pixel intensities of the immunoreactive bands were quantified using the percentage adjusted volume feature of Quantity One 5.6.4 software (Bio-Rad Laboratories, Hercules, CA, USA). Data were expressed as the ratio of the band intensity of the protein of interest to the loading control protein band (lamin B for nuclear extracts and β-actin for total extracts).

### Organotypic hippocampal slice cultures (OHSCs)

Organotypic hippocampal slice cultures were prepared as previously described with minor modifications [39]. In brief, slice cultures were prepared from 2 to 3 days old C57BL/6 J mouse pups under sterile conditions. After decapitation, the brains were removed and the hippocampi from both hemispheres were acutely isolated on ice cold serum-free Hank's Balanced Salt Solution (HBSS, PAA, Piscataway, USA), supplemented with 0.5% glucose (Sigma-Aldrich) and 15 mM HEPES (PAA). Isolated hippocampi were cut into 350-375 µM thick slices using a tissue chopper (McIlwain, MickleLab, United Kingdom) and were transferred to 0.4 µM culture plate inserts (Millipore). Four to six slices per insert, were placed in six-well plates containing 1.2 ml of culture medium per well. Culture medium (pH 7.2) consisted of 0.5× minimum essential medium (MEM, Invitrogen) containing 25% heat-inactivated horse serum (PAA), 25% BME basal medium without glutamate (Invitrogen), 2 mM glutamax and 0.65% glucose (Sigma-Aldrich). The slice cultures were kept at 35°C in a humidified atmosphere (5% CO_2_). Culture medium was refreshed the first day after preparation and every consecutive 2 days.

#### Depletion of microglia from slice cultures

Depletion of microglia from hippocampal slices was carried out as previously reported [39], [40]. In brief, slices were placed on culture plate inserts and incubated with approximately 0.5 mg/ml Lip-CL solution (1∶10 liposome dilution in standard culture medium) (ClodronateLiposomes.org, Amsterdam, The Netherlands) for 24 h at 35°C. Subsequently, slice cultures were carefully rinsed in PBS (35°C) to wash away residual liposomes and placed on fresh culture medium. After depletion the medium was refreshed every 2 days.

#### Excitotoxic lesion and treatment in hippocampal slices

After 7 days in vitro (DIV), slices were placed in medium containing 10 µM N-methyl-D-aspartic acid (NMDA, Sigma) for 4 h. For diazoxide treatment, different concentrations of the drug (200, 100, 10, 1 µM) were placed 30 min before NMDA lesion. Subsequently, the medium was replaced with standard culture medium containing different concentrations of diazoxide for 24 h after the NMDA damage. To quantify neuronal cell death in response NMDA-induced excitotoxicity, slice cultures were incubated with 5 µg/ml propidium iodide (PI, Sigma-Aldrich), during and after NMDA insult. After 24 h NMDA treatment, slices were rinse twice in PBS and fixed with 4% PFA overnight and stained with NeuN to localize the neuron population colocalizating with PI staining. Confocal images of neuronal cell layers were acquired at 10X magnification. For cell death quantification, 6 slices per condition were used in each experiment. Cell death in the CA1 layer was quantified by total PI uptake (fluorescent intensity) using ImageJ software and was expressed as percentage of PI uptake compared with untreated control slices.

### Organotypic cerebellar slice cultures (OCSCs)

The cerebellar slices cultures were prepared as previously reported [41], [42]. Briefly, parasagittal slices of postnatal at days 7-9 (P7-P9) mouse cerebellum were cut at 350 µm using a tissue chopper (McIlwain) and were transferred to 0.4 μM culture plate inserts (Millipore). Cerebellar slices were cultured in 50% basal medium with Earle's salts, 25% Hank's buffered salt solution, 25% horse serum, 5 mg/ml glucose (all from Invitrogen) at 37°C and 5% CO_2_. Three slices were placed in each culture plate insert. Culture medium was refreshed the first day after preparation and every consecutive 2 days. For LPS induced demyelination, medium was removed after 7 DIV and fresh medium with 15 µg/mL LPS was added for 24 h. For diazoxide treatment, 10-1 µM diazoxide was added 30 min before LPS treatment and during 24 h. After that, slices were rinse in PBS and fixed with 4% PFA overnight for immunostaining. OCSCs were stained for heavy neurofilament (NFH) and myelin (Myelin Basic Protein, MBP) to quantify demyelination. Confocal microscopy was used to obtain stacks of images at 2 µm intervals at 63X magnification. A total of 10 confocal z-stacks were done for each condition per experiment. An imageJ macro designed to calculate NFH intensity without MBP colocalization was used to quantify demyelination. Axon demyelination was quantified as percentage of NFH without colocalization with MBP from total NFH staining.

### Immunostaining for organotypic cultures

After fixation, organotypic slices were cut from the insert and placed in a 24 well plate. Slices were blocked for 1 h with PBS+10% NGS+0.3% Triton X-100 (Sigma-Aldrich) at 4°C. Primary and secondary antibody incubations were done overnight at 4°C in PBS+1% NGS+0.3% Triton X-100. After incubations, slices were washed three times in PBS. Finally, slices were mounted on slides and cover slipped using ProLong Gold (Invitrogen). The following primary antibodies were used: NeuN (mouse monoclonal, 1:800, Millipore); Neurofilament H (NFH, mouse monoclonal, 1:800, Abcam) and myelin basic protein (MBP, rabbit polyclonal, 1:400, Abcam). Secondary antibodies were Alexa Fluor 488 or Alexa Fluor 568 conjugated anti-mouse or anti-rabbit from Molecular Probes. Imaging was carried out using Zeiss LSM or Leica SP5 Spectral confocal microscope.

### TNF-α and IL-6 determination

The amount of TNF-α and IL-6 released into the culture media from OCSCs was determined using an Enzyme-linked immunosorbent assay (ELISA) kit specific for mouse TNF-α (Mouse TNF-α Ready-SET-Go!®) and for mouse IL-6 (Mouse IL-6 Ready-SET-Go!®) (both from eBioscience, San Diego, CA, USA) according to the manufacturer’s instructions.

### Statistical Analysis

Data were expressed as the mean ± SEM unless specified. Statistical analysis of cell treatments was carried out using one-way ANOVA, using repeated measures ANOVA when appropriate, followed by Newman-Keuls post test when three or more experimental groups were compared and Student’s *t*-test when only two groups were compared. Values of p<0.05 were considered statistically significant.

## Results

### Diazoxide improves NSC-34 motoneuron survival after excitotoxic, oxidative and inflammatory damage

**Figure 1:**
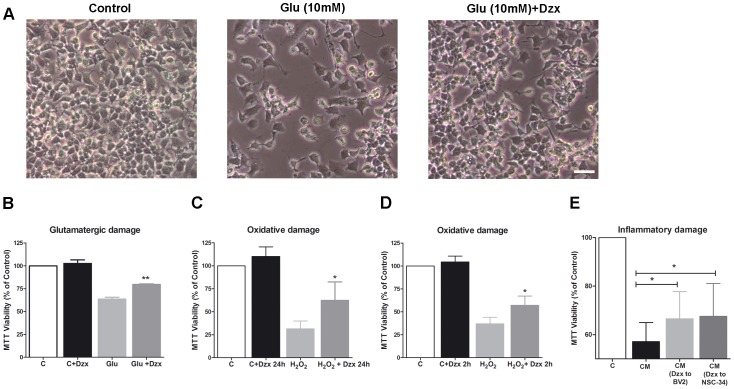
Diazoxide prevents neuronal damage after different neurotoxic insults. Representative images of differentiated NSC-34 motoneuron cells line after glutamate damage and diazoxide treatment (A). Diazoxide inhibited glutamate mediated cell death by 24 h in differentiated NSC-34 motoneuron cell line (B). Diazoxide inhibited H_2_O_2_ mediated cell death by 24 h (C) and also when used as pretreatment during 2 h (survival analysis performed 24 h after toxic insult) (D). Diazoxide inhibited inflammatory BV2 microglial mediated cell death by 24 h in NSC-34 motoneuron. Conditioned Medium (CM): Neurons damaged with activated BV2 medium. CM (Dxz to NSC-34): Neurons treated with diazoxide and damaged with activated BV2 medium afterwards. CM (Dzx to BV2): Neurons damaged with diazoxide treated BV2 activated medium (E). Diazoxide treatment 100 µM. Results expressed as mean ± SEM. n≥4 experiments. *: p < 0.05. Scale bar =  30 µm

To examine the protective effect of diazoxide, we tested its effects in NSC-34 motoneurons subject to different neurotoxic insults. When diazoxide effects were tested during glutamatergic insult, results showed that the compound protected motoneuron cells against glutamate toxicity (Figure 1A), producing a significant increase of cell viability (16±1% *vs* untreated damaged cells, Figure 1B). Second, H_2_O_2_ was used to produce exogenous ROS damage (viability loss 68±8%, Figure 1C-D). Diazoxide produced a significant increase of NSC-34 viability after H_2_O_2_ challenge in comparison to untreated cells when the treatment was maintained during 24 h (31±13%, Figure 1C) but also when the drug was removed just after the H_2_O_2_ damage (17±5%, Figure 1D). Finally, when diazoxide was tested during inflammatory damage, treatment produced a significant increase of NSC-34 viability in both paradigms: by protecting neurons against BV2 conditioned medium (CM) and by decreasing BV2 neurotoxicity (10±3% and 12±5% increase *vs* untreated condition, Figure 1E). These results demonstrate that diazoxide could protect NSC-34 neurons against the main sources of neurodegenerative damage.

### Neuroprotective effects of diazoxide in mouse OHSCs are maintained after microglia depletion

**Figure 2:**
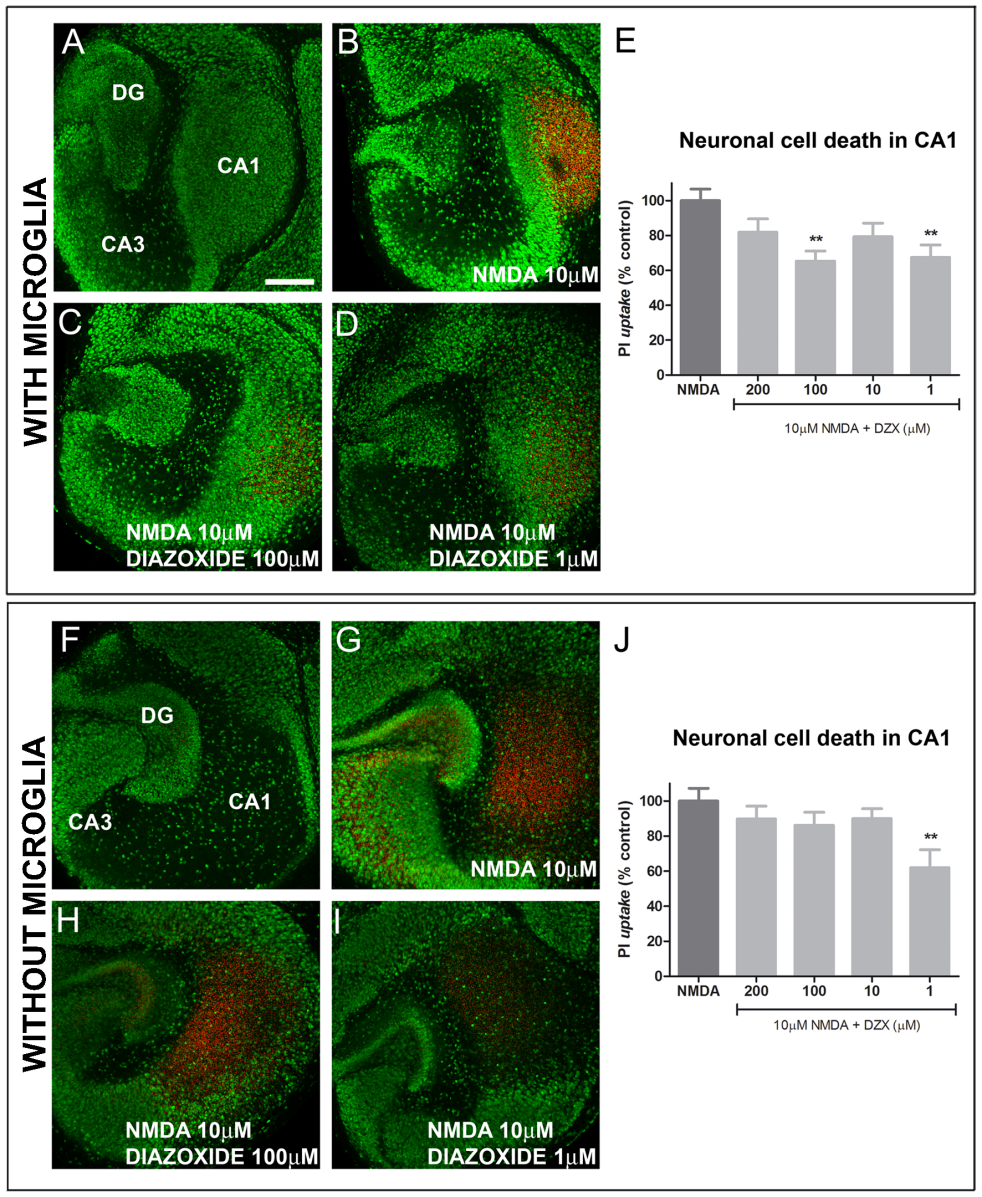
Diazoxide effects in NMDA-induced neurodegeneration in hippocampal slice culture. At 7 DIV, treatment with 10 µM NMDA for 4 h induced a region-specific increase neuronal cell death of the CA1 layer compared to undamaged slices, as determined as PI uptake (PI, red) in colocalization with the neuronal nuclear marker NeuN (green) (A, B). Treatment 30 min before NMDA lesion with diazoxide prevented cell death, significantly at 100 µM but also at lowest dose 1 µM (C, D). Upon quantification, results showed a significant decrease at 100 µM (65±5% in PI uptake compared to control) and 1 µM (67±7% in PI uptake compared to control (E). When microglia was removed from the slices, cell protection remained at the lowest dose 1 µM (62±10% in PI uptake compared to control) (F-J). Data are a summary of four to seven individual experiments with 6 slices per conditions analyzed in each experiment. Results are shown as mean ± SEM. * p<0.05, ** p<0.01. Scale bar 300 µm

To examine the effects of diazoxide in a model of neuronal degeneration induced by NMDA excitotoxicity, OHSCs were used. After 7 DIV, the neuronal layers CA1, CA3 and dentate gyrus (DG) were well preserved and neuronal cell death was minimal (<1%, Figure 2A). Treatment with 10 µM NMDA for 4 h induced a region-specific neuronal cell death in CA1 layer with minor affectation of CA3 layer and DG (Figure 2B). When OHSCs were pretreated for 30 min with different concentrations of diazoxide, hippocampal damage, measured as PI uptake, was reduced (Figure 2C, D). A significant prevention in cell death was observed at 100 µM diazoxide (65±6% in PI uptake compared to control) but also at the lowest dose tested, 1 µM (67±7% in PI uptake compared to control) (Figure 2E).

In order to elucidate whether the neuroprotective effect of diazoxide in OHSCs was mediated by microglia, microglial cells were depleted from mouse slice cultures using liposomes-encapsulated clodronate (Lip-CL). Lip-CL has been shown to successfully deplete microglia from mouse organotypic slice cultures, without affecting other cell-types [39], [43] or hippocampal architecture (Figure 2F). As previously described [40], neuronal cell death in microglia-depleted slice culture was enhanced in response to 10 µM NMDA compared to normal OHSC (Figure 2G). When slices were pretreated with different doses of diazoxide (200-1 µM), the protective effect at the high doses was abolished (Figure 2H) but protection remained at the lowest dose 1 µM (62±10% PI uptake compared to control, Figure 2I, J). These results indicate that the protective effects of diazoxide observed could be partially mediated by microglia.

### Diazoxide protects against demyelination in LPS-neuroinflammation model in organotypic cerebellar cultures

**Figure 3:**
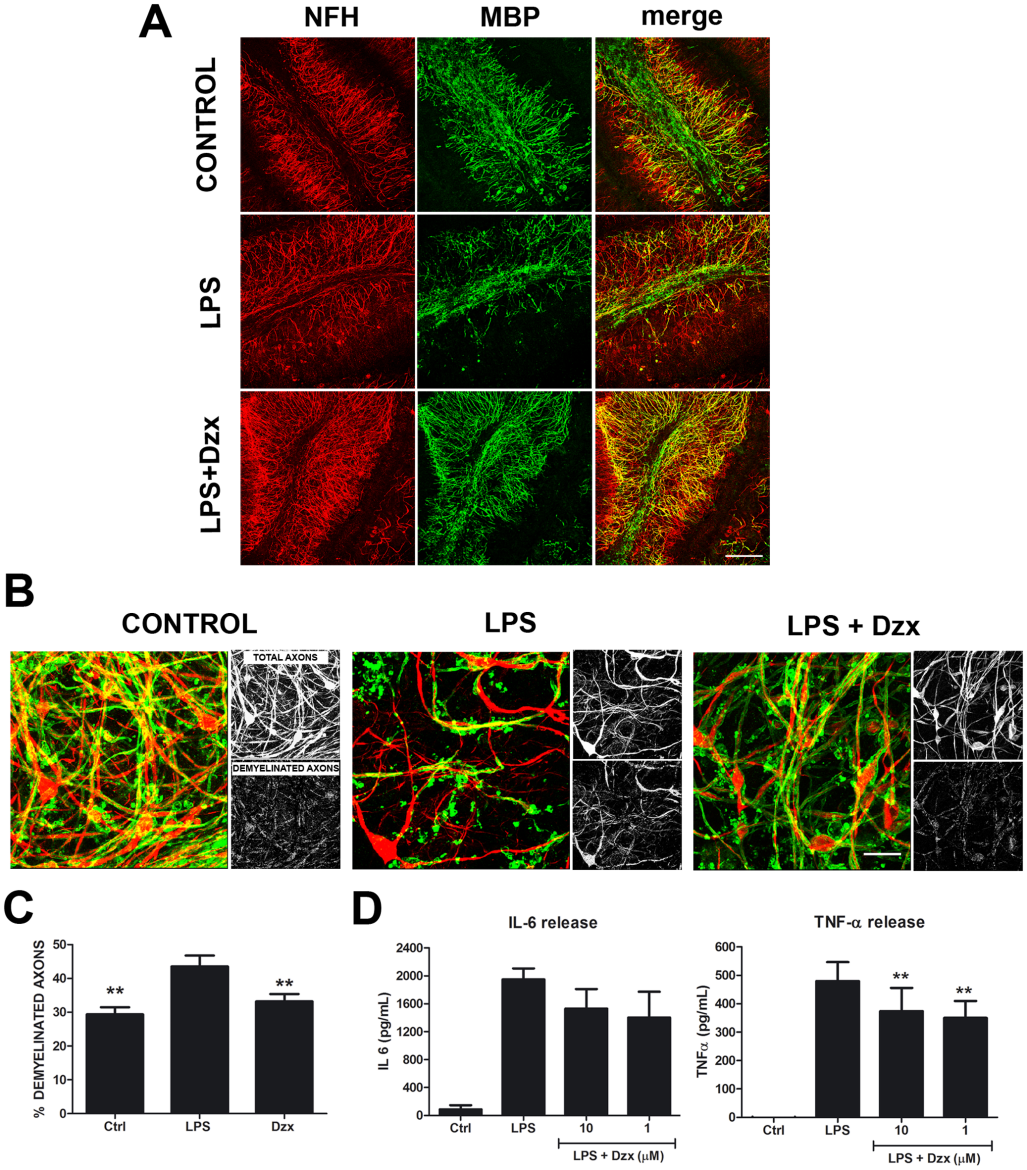
Diazoxide prevents LPS-induced demyelination in organotypic cerebellar culture. Cerebellar slices were treated with 15µg/ml LPS to induce demyelination as shown with heavy neurofilament (NFH, red) and myelin basic protein (MBP, green) immunostaining. Representative images from untreated, LPS and LPS+10 µM diazoxide treated cerebellar slices demonstrated that treatment preserved MBP staining and prevented myelin debris accumulation (A). Higher magnifications of axons from untreated, LPS and LPS+10 µM diazoxide treated cerebellar slices are shown. Small panels show total NFH intensity (Total axons) and NFH staining without colocalization with MBP staining (Demyelinated axons) (B). Axon demyelination was quantified as percentage of NFH without colocalization with MBP from total NFH staining. Upon quantification of axon demyelination, results showed that diazoxide reduced axonal myelin loss when compared to LPS treated slices (33±2% *vs* 44±3%, respectively) (C). 10 and 1 µM diazoxide also reduced TNF-α and IL-6 release derived from LPS stimulation in cerebellar cultures. This decrease was statistical significant for TNF-α (480±67 pg/mL from LPS treated slices *vs* 374±82 pg/mL and 350±60 pg/mL from 10 µM and 1 µM diazoxide LPS treated slices, respectively) (D). Results are shown as mean ± SEM. ** p<0.01. Scale bar 200 µm for A. Scale bar 50 µm for B

To explore whether diazoxide prevents demyelination and axonal damage, a LPS-mediated demyelination model in cerebellar organotypic cultures was used. As previously reported, treatment with 15 µg/mL LPS for 24 h promotes microglial activation and consequent demyelination and axonal damage [41]. In our cultures, slices treated with 15 µg/mL LPS for 24 h showed a decrease in MBP intensity and an increase of myelin debris (Figure 3A). When slices were treated with 10 µM diazoxide during injury, a better preservation of myelin structure was observed (Figure 3A). Lower diazoxide concentrations also showed positive effects on myelin preservation (data not shown). At higher magnification, demyelination was observed in LPS treated slices (Figure 3B). Demyelinated axon quantification, measured as heavy neurofilament (NFH) intensity without colocalization with myelin (MBP signal), showed a 15% increase of demyelinated axons in LPS treated slice compared to control (44±3% *vs* 29±2%, respectively). Treatment with diazoxide significantly prevents myelin loss induced by LPS (44±3% from LPS treated slices *vs* 33±2% from diazoxide LPS treated slices) (Figure 3C). In order to prove whether pro-inflammatory signals derived from LPS treatment were modified by diazoxide, IL-6 and TNF-α content in the medium was analyzed. Results showed that these inflammatory mediators were decreased in treated slices (Figure 3D). This decrease was statistical significant for TNF-α (480±67 pg/mL from LPS treated slices *vs* 374±82 pg/mL and 350±60 pg/mL from 10 µM and 1 µM diazoxide LPS treated slices, respectively). Taken together, these results show the neuroprotective effects of diazoxide in pro-inflammatory demyelinating insults.

### Diazoxide promotes nuclear Nrf2 translocation and decreases oxidative cell damage at low doses in NSC-34 motoneuron

**Figure 4:**
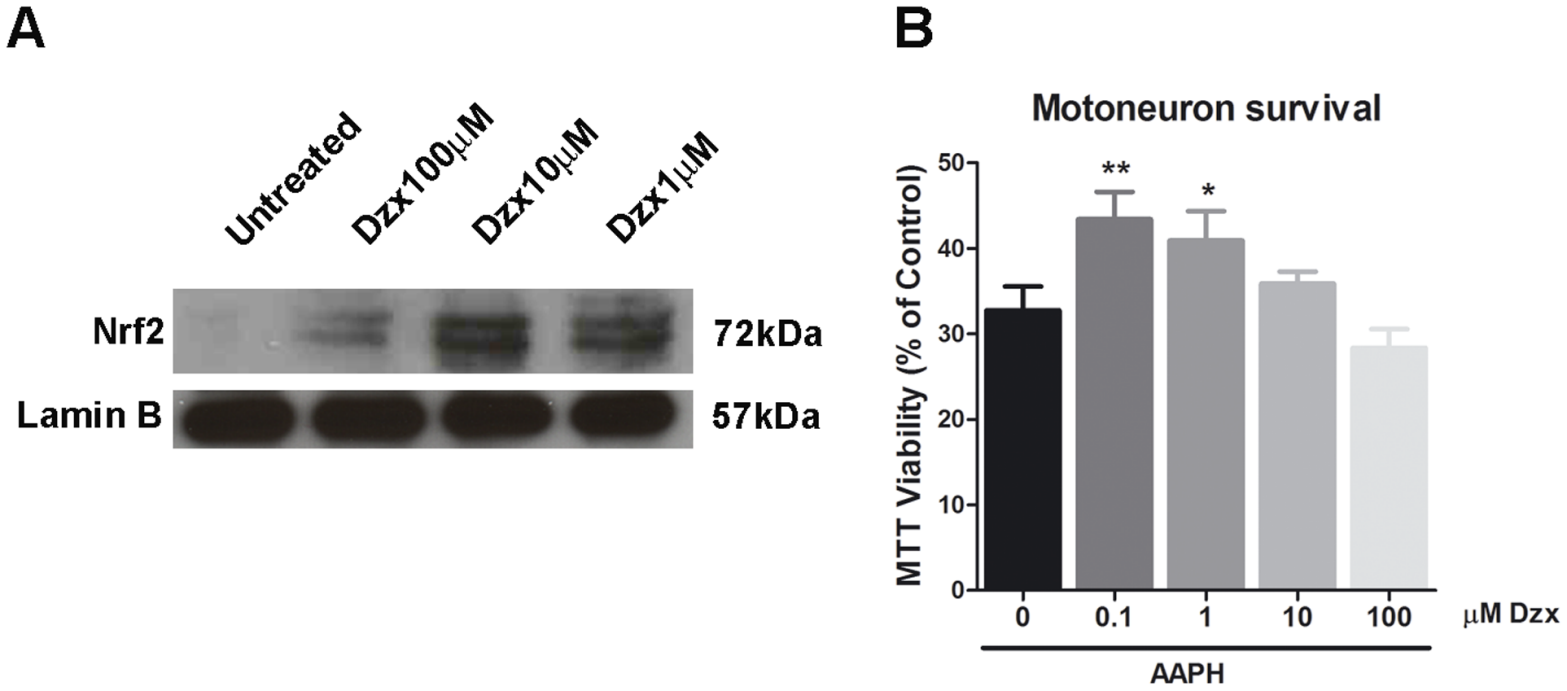
Diazoxide increases Nrf2 nuclear translocation in NSC-34 motoneurons and prevents endogenous oxidative damage. Western blot showed an increase of Nrf2 signaling in the nuclear extracts from NSC-34 neurons treated with different doses of diazoxide for 24 h. The higher increase of Nrf2 was found at lower doses (10 and 1 µM) (A). Cell viability of NSC-34 cells was measured after 24 h AAPH oxidative stress activation and results demonstrated that diazoxide treatment effectively ameliorates cell viability at low doses (B). Results expressed as mean ± SEM. n≥4 experiments. *: p < 0.05, ** p<0.01. Scale bar  =  30 µm

To elucidate whether pharmacological activation of anti-oxidative pathways was involved in the direct neuroprotection exerted by diazoxide in NSC-34 neurons, we performed an analysis of Nrf2 expression 24 h after diazoxide treatment. Immunoblotting analysis demonstrated an important increase of Nrf2 signal in nuclear fraction of NSC-34 cells treated with diazoxide. Interestingly, the increase was higher at low doses of the compound (Figure 4A). To explore whether the increase of Nrf2 nuclear translocation on NSC-34 at low doses was correlated with an increase of cell viability in presence of endogenous oxidative stress, cell survival was analyzed after AAPH exposure. In concordance with the previous results, diazoxide treatment significantly ameliorated cell survival at low doses (1 µM and 0.1 µM) (Figure 4B, 8.1±2.7% and 10.7±1.6%, respectively)

### Diazoxide treatment promotes Nrf2 translocation and causes neuroprotection in EAE mice spinal cord

**Figure 5:**
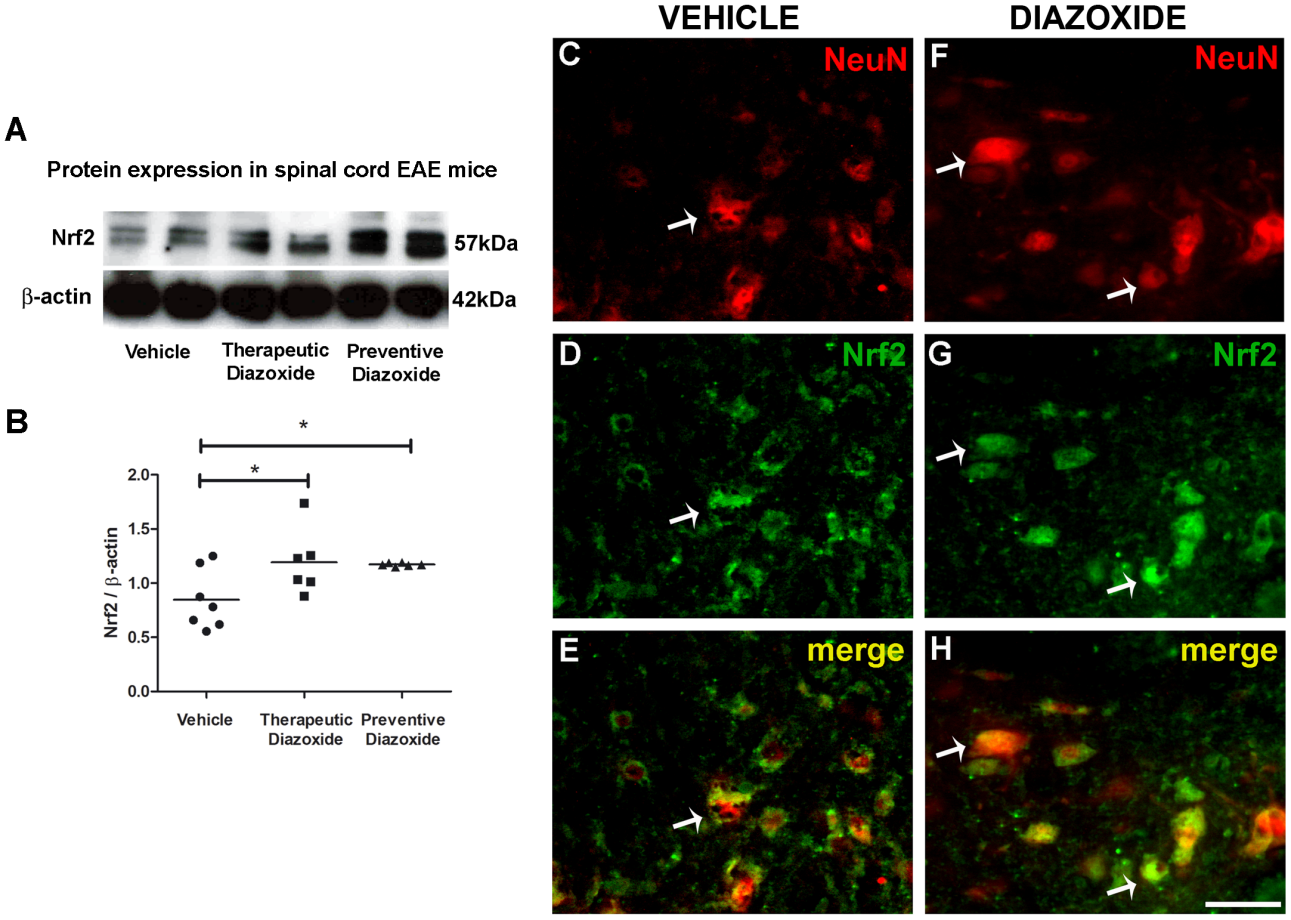
Oral diazoxide administration increases Nrf2 expression and neuronal nuclear translocation in spinal cord of EAE mice. Western blotting of spinal cord total protein showed an increase of Nrf2 immunostain in protein extracts from 0.8/Kg/day oral diazoxide treated EAE mice (either therapeutically and preventively) when compared to vehicle EAE mice (A). Upon quantification, results showed that this increase was significant (B). Double immunofluorescent staining for NeuN (red, C, F) and Nrf2 (green, D, G) of vehicle EAE and 0.8 mg/Kg/day oral diazoxide treated EAE mice showed better NeuN signal preservation (C *vs* F) and Nrf2 nuclear translocation (E, D *vs* G, H; white arrows) in the spinal cord ventral horn. n≥6 animal/group. *: p<0.05. Scale bar  =  50 µm

Nrf2 levels were also measured by western blot using total spinal cord homogenates from EAE animals treated with oral diazoxide (preventively and therapeutically) and control mice. Results showed a significant increase of Nrf2 immunostaining in diazoxide treated animals when compared to vehicle mice (Figure 5A, B). Next, histological observation of diazoxide treated and control spinal cords from EAE animals were performed. Control EAE mice showed signs of neurodegeneration, characterized by nuclear pigmentation, loss of tissue integrity and decrease of NeuN immunoreactivity, a classic marker for functional mature neurons (Figure 5C). Diazoxide treated animals showed higher NeuN stain and higher preservation of the grey matter than vehicle control EAE animals (Figure 5C, F). Double immunofluorescence analysis of diazoxide treated animals showed a nuclear translocation for Nrf2 in NeuN positive motoneuron, demonstrating an activation of Nrf2 anti-oxidative pathway (Figure 5D, E *vs* Figure 5G, H, white arrows). Thus, activation of endogenous antioxidative self-defense could be involved in beneficial effect of diazoxide in EAE mice.

## Discussion

The use of selective agonists of K_ATP_ channel has been found to protect cell death in many cell types against excitotoxicity, oxidative stress or inflammatory insult [44]–[46] and to produce beneficial outcomes in different animal models of neurodegenerative pathologies [26], [30], [47]. We have previously demonstrated that diazoxide ameliorates disease progression in a murine model of MS, decreasing axonal loss and preventing neuron integrity in the spinal cord of EAE mice [30]. We showed that diazoxide could mediate an inhibition of microglial pro-inflammatory behavior that could be beneficial for EAE clinical course. Although these studies suggest that diazoxide has a neuroprotective effect in EAE, it is not clearly understood whether it is a direct effect to neurons or an indirect consequence caused by an alteration in neuronal environment. Here, we demonstrated that diazoxide preserves motoneuron viability against excitotoxic, oxidative and inflammatory neurodegenerative insults. When we study neuroprotection in a more complex system where intercellular relationships and the histoarchitecture are preserved, like the organotypic hippocampal cultures, we found that diazoxide treatment preserve NMDA-induced cell death even at low concentrations. Moreover, when we depleted microglia from these cultures we observed that the effect was sustained at the lowest dose, suggesting a direct neuroprotective effect independent of microglial cells. These results also suggest that diazoxide could have different mechanisms of action that causes neuroprotection depending of the concentration of drug. Although further experiments would be needed to better understand which exact cellular pathways are involved, low doses of diazoxide have been reported to be selective for the activation of mito-K_ATP_ channels [48], [49]. Garlid and coll. compared reconstituted cardiac mito-K_ATP_ with reconstituted cardiac sarcolemmal K_ATP_ (sarc-K_ATP_) channels and showed that mito-K_ATP_ channel is about 2000 times more sensitive to diazoxide than sarc-K_ATP_ channels, indicating that two distinct receptor subtypes coexist within the myocytes [50]. Other authors found that interaction between mito-K_ATP_ channel and protein kinase C epsilon (εPKC) during ischemic preconditioning (IPC) induction preserves the hippocampal CA1 region after ischemia. Interestingly, these results showed that only low concentrations of diazoxide were neuroprotective [51]. Several studies confirmed that the protection towards neurons conferred by diazoxide is mainly mediated by mito-K_ATP_ [52]–[54], but, unfortunately, the exact mechanism and structure of mito-K_ATP_ itself is still controversial [55], [56]. In this way, our results suggest that diazoxide has a direct effect on neurons and, as this is produced at low doses, this effect could be through activation of mito-K_ATP_ channels.

It has been shown that mito-K_ATP_ activation by diazoxide prevents neuronal oxidative stress and excitotoxic cell death in part by reducing intracellular ROS level [46]. Moreover, it is reported that diazoxide, acting at mitochondrial level, can activate defensive and anti-apoptotic mechanisms [8]. Among other pathways, Nrf2 transcription factor has been postulated as one of the main components of cell self-defense against oxidative damage and mitochondrial dysfunction [57]–[60]. To maintain a physiological redox balance, cells are equipped with a wide variety of endogenous antioxidant enzymes. Production of these cytoprotective enzymes is induced upon exposure to ROS via a mechanism regulated at the transcriptional level [61]. Genes that code for proteins involved in ROS detoxification share a common promoter element, called the antioxidant response element (ARE). ARE-mediated gene activation is coordinated by Nrf2, which, upon exposure to electrophiles or ROS, translocates to the nucleus and activates antioxidant enzyme gene transcription [62], [63]. Here, we demonstrated that low doses of diazoxide increase Nrf2 nuclear translocation in neurons. After neuron exposure to endogenous oxidative stress promoted by AAPH, diazoxide treatment exerts neuroprotection, but only at low doses, in concordance with the concentrations observed to promote Nrf2 activation. These results suggest that diazoxide neuroprotection could be partially due to the activation of endogenous antioxidant pathways mediated at mitochondrial level. Moreover, when Nrf2 activation was studied in diazoxide EAE treated animals, an increase of this nuclear factor was observed in spinal cord. In EAE animals, protective effects of different exogenous antioxidants have been described [64], [65]. However, another alternative strategy to inhibit the detrimental effects of ROS is through the induction of endogenous antioxidants enzymes [66]. In this way, as we just mentioned, the translocation of Nrf2 to the nucleus in neurons of EAE animals treated with diazoxide could be the cause of Nrf2 endogenous anti-oxidant pathway activation, and this might produce a neuroprotective effect. Interestingly, in this way, Nrf2 activation has been described as the key mechanism of action for BG-12, a novel therapeutic compound for the treatment of MS [32], [33], [67]. Finally, when we study whether diazoxide could have a protective effect in LPS induced demyelination, we observed that the treatment can also prevent myelin loss in cerebellar cultures. Since diazoxide has been reported to promote oligodendrocyte precursor cell proliferation and myelination [68] and inhibit inflammatory glial reactivity [30], more studies would be needed to clarify the possible role of diazoxide in myelin repair. Taken together these results suggest that diazoxide is a promising neuroprotective agent for the protection of oxidative stress-induced damage and dysfunction in neurodegenerative and inflammatory diseases such MS.
