# Correction: K_ATP_ Channel Opener Diazoxide Prevents Neurodegeneration: A New Mechanism of Action via Antioxidative Pathway Activation

**DOI:** 10.1371/annotation/0e045706-ea24-41db-be90-27d1cbcd35b1

**Published:** 2013-09-19

**Authors:** Noemí Virgili, Pilar Mancera, Blanca Wappenhans, Georgina Sorrosal, Knut Biber, Marco Pugliese, Juan F. Espinosa-Parrilla

The fifth author, Knut Biber, should also have the affiliation "Department of Neuroscience, University Medical Center Groningen, The Netherlands"

There was an error in the Figure 4 legend. The correct version of the legend is available below.

**Figure 4. Diazoxide increases Nrf2 nuclear translocation in NSC-34 motoneurons and prevents endogenous oxidative damage.**

Western blot showed an increase of Nrf2 signaling in the nuclear extracts from NSC-34 neurons treated with different doses of diazoxide for 24 h. The higher increase of Nrf2 was found at lower doses (10 and 1 µM) (A). Cell viability of NSC-34 cells was measured after 24 h AAPH oxidative stress activation and results demonstrated that diazoxide treatment effectively ameliorates cell viability at low doses (B). Results expressed as mean ± SEM. n≥4 experiments. *: p < 0.05, ** p<0.01. 

